# Crystal structure of 1-[2-(di­ethyl­aza­n­ium­yl)eth­yl]-3-methyl­imidazolium tetra­chlorido­cuprate(II)

**DOI:** 10.1107/S2056989015006799

**Published:** 2015-04-18

**Authors:** Gerhard Laus, Volker Kahlenberg, Herwig Schottenberger

**Affiliations:** a University of Innsbruck, Faculty of Chemistry and Pharmacy, Innrain 80, 6020 Innsbruck, Austria; b University of Innsbruck, Institute of Mineralogy and Petrography, Innrain 52, 6020 Innsbruck, Austria

**Keywords:** crystal structure, copper(II) complex, tetra­chlorido­cuprate, 1-[2-(di­ethyl­aza­nium­yl)eth­yl]-3-methyl­imidazolium dication, hydrogen bonding

## Abstract

The title compound, (C_10_H_21_N_3_)[CuCl_4_], is composed of one 1-[2-(di­ethyl­aza­nium­yl)eth­yl]-3-methyl­imidazolium dication and a tetra­chlorido­cuprate(II) dianion. The anion adopts a distorted tetra­hedral geometry. Bifurcated interionic N—H⋯Cl hydrogen bonds and several C—H⋯Cl contacts are observed, leading to a layer-like arrangement of the components parallel to (100).

## Related literature   

For structures of related tetra­chlorido­cuprates(II), see: Russell & Wallwork (1969 ▸); Główka & Gilli (1989 ▸); Choi *et al.* (2002 ▸); Sun & Qu (2005 ▸); Elangovan *et al.* (2007*a* ▸,*b* ▸); Strasser *et al.* (2007 ▸). For details of the synthesis, see: Laus *et al.* (2012 ▸); Håkansson & Jagner (1990 ▸).
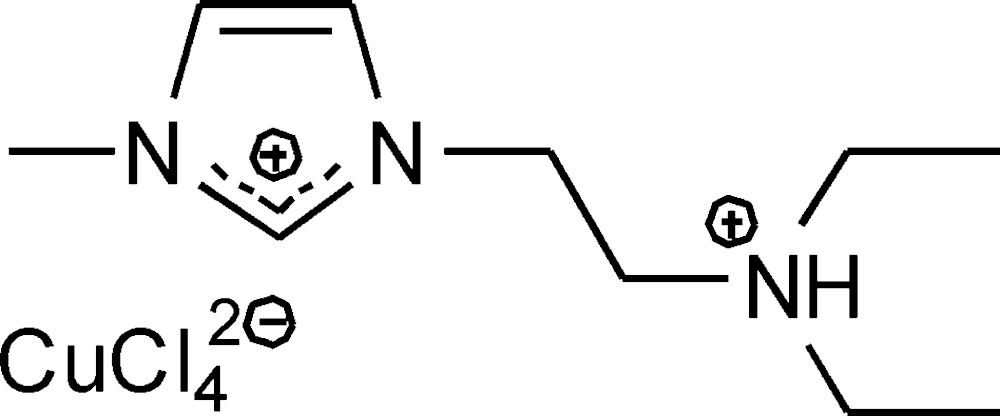


## Experimental   

### Crystal data  

(C_10_H_21_N_3_)[CuCl_4_]
*M* *_r_* = 388.64
Monoclinic, 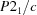

*a* = 17.0041 (8) Å
*b* = 7.1161 (6) Å
*c* = 14.4143 (7) Å
β = 112.956 (6)°
*V* = 1606.04 (17) Å^3^
*Z* = 4
Mo *K*α radiation
μ = 2.01 mm^−1^
*T* = 173 K
0.20 × 0.16 × 0.12 mm

### Data collection  

Oxford Diffraction Gemini-R Ultra diffractometer
Absorption correction: multi-scan (*CrysAlis PRO*; Oxford Diffraction, 2010 ▸) *T*_min_ = 0.875, *T*_max_ = 1
10390 measured reflections
2991 independent reflections
2361 reflections with *I* > 2σ(*I*)
*R*_int_ = 0.045

### Refinement  

*R*[*F* ^2^ > 2σ(*F* ^2^)] = 0.032
*wR*(*F* ^2^) = 0.056
*S* = 0.98
2991 reflections
166 parameters
H-atom parameters constrained
Δρ_max_ = 0.34 e Å^−3^
Δρ_min_ = −0.34 e Å^−3^

### 

Data collection: *CrysAlis PRO* (Oxford Diffraction, 2010 ▸); cell refinement: *CrysAlis PRO*; data reduction: *CrysAlis PRO*; program(s) used to solve structure: *SIR2002* (Burla *et al.*, 2003 ▸); program(s) used to refine structure: *SHELXL97* (Sheldrick, 2008 ▸); molecular graphics: *ORTEP-3 for Windows* (Farrugia, 2012 ▸); software used to prepare material for publication: *SHELXL97*.

## Supplementary Material

Crystal structure: contains datablock(s) I, global. DOI: 10.1107/S2056989015006799/ff2135sup1.cif

Structure factors: contains datablock(s) I. DOI: 10.1107/S2056989015006799/ff2135Isup2.hkl

Supporting information file. DOI: 10.1107/S2056989015006799/ff2135Isup3.mol

Supporting information file. DOI: 10.1107/S2056989015006799/ff2135Isup4.cml

**Fig. 1.**
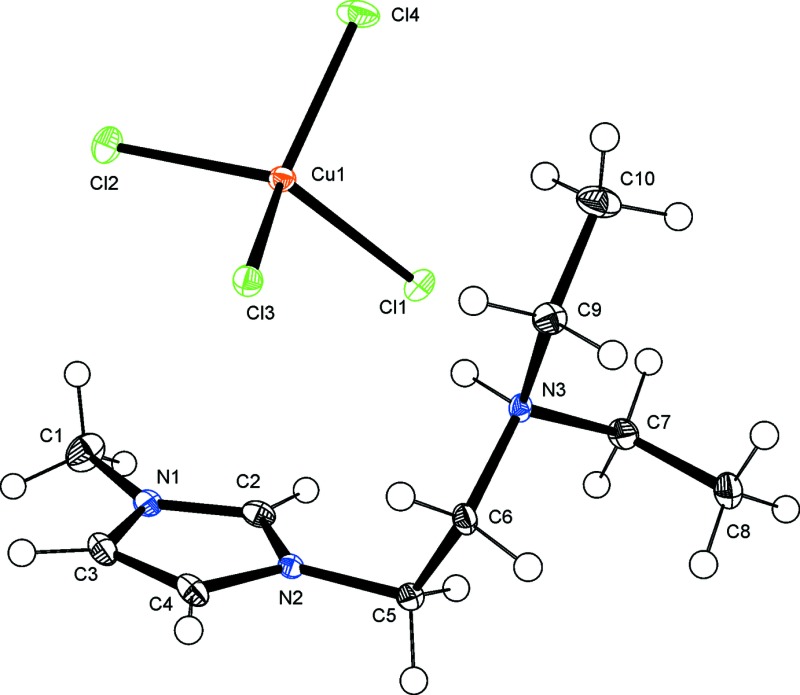
Ion pair of the title compound, with atom labels and 50% probability displacement ellipsoids for non-H atoms.

. DOI: 10.1107/S2056989015006799/ff2135fig1.tif
Ion pair of the title compound, with atom labels and 50% probability displacement ellipsoids for non-H atoms.

**Fig. 2.**
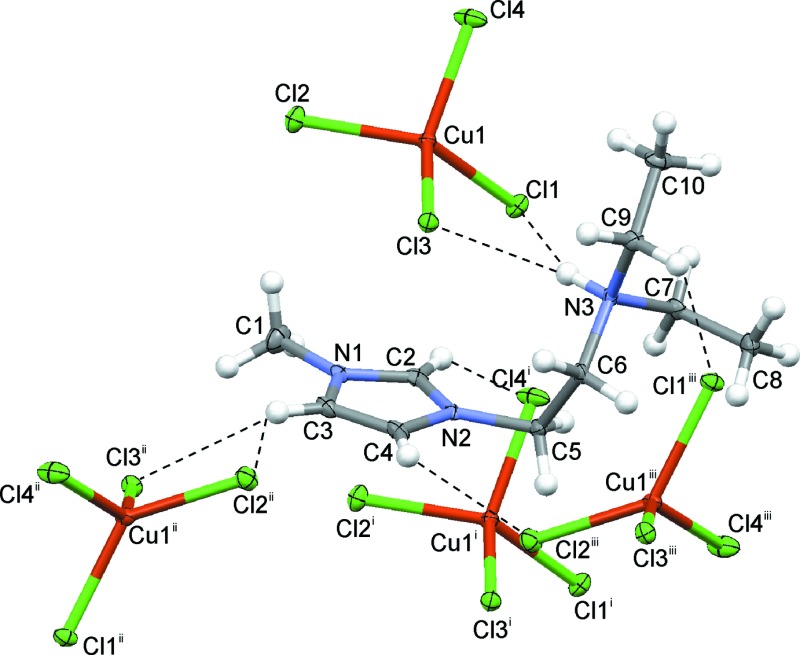
Interionic contacts in the crystal structure of the title compound. Symmetry codes: (i) x, -1 + y, z; (ii) –x, -1/2 + y, 3/2–z; (iii) x, 1/2–y, 1/2 + z.

Click here for additional data file.x y z x y z x y z . DOI: 10.1107/S2056989015006799/ff2135fig2.tif
Inter­ionic contacts in the crystal structure of the title compound. Symmetry codes: (i) *x*, −1 + *y*, *z*; (ii) –*x*, −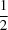 + *y*, 3/2–*z*; (iii) *x*, 1/2–*y*, 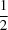 + *z*.

CCDC reference: 1057934

Additional supporting information:  crystallographic information; 3D view; checkCIF report

## Figures and Tables

**Table 1:** Selected bond lengths ()

Cu1Cl4	2.2267(7)
Cu1Cl3	2.2447(6)
Cu1Cl2	2.2456(8)
Cu1Cl1	2.2644(7)

**Table 2:** Hydrogen-bond geometry (, )

*D*H*A*	*D*H	H*A*	*D* *A*	*D*H*A*
N3H3*N*Cl1	0.93	2.29	3.134(2)	150
N3H3*N*Cl3	0.93	2.79	3.399(2)	124
C2H2Cl4^i^	0.95	2.66	3.480(3)	145
C3H3Cl2^ii^	0.95	2.75	3.423(3)	128
C3H3Cl3^ii^	0.95	2.77	3.537(3)	138
C4H4Cl2^iii^	0.95	2.84	3.608(3)	139
C9H9*A*Cl1^iii^	0.99	2.78	3.617(3)	143

Symmetry codes: (i) 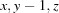; (ii) 
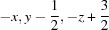
; (iii) 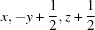.

## supplementary crystallographic information

### S1. Comment

1-[2-(Diethylazaniumyl)ethyl]-3-methylimidazolium ions in deprotonated form can act as bidentate ligands (Laus *et al.*, 2012). In this work, it is shown that they are also capable of coordinating in protonated form. The structure of the ion pair of 3-(2-(diethylammonio)ethyl-1- methylimidazolium tetrachlorocuprate (II) is shown in Figure 1. The anion adopts a distorted tetrahedral geometry. The Cl—Cu—Cl angles range from 97° to 134°. The heterocyclic rings of the cation are oriented parallel to the (3 7 2) and (3 7 2) planes with an interplanar angle of 32.5°. The side chain is twisted out of the heterocyclic ring plane. In the crystal structure, the cations and anions are linked by N—H···Cl and C—H···Cl hydrogen bonds (Figure 2).

### S2. Experimental

The title compound, (C_10_H_21_N_3_)CuCl_4_, was obtained from the reaction of bis-(1-(2-(diethylamino)ethyl)-3-methylimidazolin-2-ylidene) di-silver(I) bis(bis(triflimide)) (Laus *et al.*, 2012) and carbonyl chlorocopper(I) (Håkansson & Jagner, 1990) in methanol under ambient conditions. This unconventional synthesis involves redox, protonation and complexation steps. Yellow-green single crystals were obtained from MeOH in modest yield. Melting point 118–120 °C.

### S3. Refinement

All hydrogen atoms were positioned geometrically and constrained to ride on their parent atoms with *U*_iso_(H) = 1.2–1.5*U*_eq_(parent atom).

### Crystal data

**Table:**

(C_10_H_21_N_3_)[CuCl_4_]	*F*(000) = 796
*M**_r_* = 388.64	*D*_x_ = 1.607 Mg m^−^^3^
Monoclinic, *P*2_1_/*c*	Mo *K*α radiation, λ = 0.71073 Å
*a* = 17.0041 (8) Å	Cell parameters from 4805 reflections
*b* = 7.1161 (6) Å	θ = 3.1–28.5°
*c* = 14.4143 (7) Å	µ = 2.01 mm^−^^1^
β = 112.956 (6)°	*T* = 173 K
*V* = 1606.04 (17) Å^3^	Fragment, yellow
*Z* = 4	0.20 × 0.16 × 0.12 mm

### Data collection

**Table:**

Oxford Diffraction Gemini-R Ultra diffractometer	2991 independent reflections
Graphite monochromator	2361 reflections with *I* > 2σ(*I*)
Detector resolution: 10.3822 pixels mm^-1^	*R*_int_ = 0.045
ω (1° width) scans	θ_max_ = 25.5°, θ_min_ = 3.1°
Absorption correction: multi-scan (*CrysAlis PRO*; Oxford Diffraction, 2010)	*h* = −20→20
*T*_min_ = 0.875, *T*_max_ = 1	*k* = −6→8
10390 measured reflections	*l* = −17→17

### Refinement

**Table:**

Refinement on *F*^2^	Primary atom site location: structure-invariant direct methods
Least-squares matrix: full	Secondary atom site location: difference Fourier map
*R*[*F*^2^ > 2σ(*F*^2^)] = 0.032	Hydrogen site location: inferred from neighbouring sites
*wR*(*F*^2^) = 0.056	H-atom parameters constrained
*S* = 0.98	*w* = 1/[σ^2^(*F*_o_^2^) + (0.0234*P*)^2^] where *P* = (*F*_o_^2^ + 2*F*_c_^2^)/3
2991 reflections	(Δ/σ)_max_ = 0.001
166 parameters	Δρ_max_ = 0.34 e Å^−^^3^
0 restraints	Δρ_min_ = −0.34 e Å^−^^3^

### Special details

**Table:**

Experimental. Empirical absorption correction using spherical harmonics, implemented in SCALE3 ABSPACK scaling algorithm.
Geometry. All e.s.d.'s (except the e.s.d. in the dihedral angle between two l.s. planes) are estimated using the full covariance matrix. The cell e.s.d.'s are taken into account individually in the estimation of e.s.d.'s in distances, angles and torsion angles; correlations between e.s.d.'s in cell parameters are only used when they are defined by crystal symmetry. An approximate (isotropic) treatment of cell e.s.d.'s is used for estimating e.s.d.'s involving l.s. planes.
Refinement. Refinement of *F*^2^ against ALL reflections. The weighted *R*-factor *wR* and goodness of fit *S* are based on *F*^2^, conventional *R*-factors *R* are based on *F*, with *F* set to zero for negative *F*^2^. The threshold expression of *F*^2^ > σ(*F*^2^) is used only for calculating *R*-factors(gt) *etc*. and is not relevant to the choice of reflections for refinement. *R*-factors based on *F*^2^ are statistically about twice as large as those based on *F*, and *R*- factors based on ALL data will be even larger.

### Fractional atomic coordinates and isotropic or equivalent isotropic displacement parameters (Å^2^)

**Table:**

	*x*	*y*	*z*	*U*_iso_*/*U*_eq_	
Cu1	0.24089 (2)	0.54432 (5)	0.77447 (2)	0.01040 (9)	
Cl1	0.33356 (4)	0.30011 (9)	0.81388 (5)	0.01280 (16)	
Cl2	0.12793 (4)	0.62361 (10)	0.63485 (5)	0.01575 (16)	
Cl3	0.17620 (4)	0.47770 (9)	0.87973 (4)	0.01224 (15)	
Cl4	0.33226 (4)	0.76469 (10)	0.76985 (6)	0.02164 (18)	
N3	0.34210 (13)	0.2088 (3)	1.03033 (15)	0.0089 (5)	
H3N	0.3191	0.2461	0.9632	0.011*	
N2	0.16575 (13)	−0.0096 (3)	0.88310 (15)	0.0089 (5)	
N1	0.08640 (14)	0.0672 (3)	0.73070 (15)	0.0118 (5)	
C9	0.35838 (17)	0.3852 (4)	1.09319 (19)	0.0130 (6)	
H9A	0.3812	0.3503	1.1654	0.016*	
H9B	0.3036	0.4519	1.0777	0.016*	
C2	0.16480 (17)	0.0163 (4)	0.79170 (19)	0.0125 (6)	
H2	0.2121	0.0012	0.7729	0.015*	
C8	0.46893 (17)	0.0322 (4)	1.15266 (19)	0.0164 (6)	
H8A	0.4892	0.1404	1.1978	0.025*	
H8B	0.5178	−0.0452	1.1564	0.025*	
H8C	0.4301	−0.0429	1.1731	0.025*	
C6	0.27576 (16)	0.0917 (4)	1.04753 (19)	0.0105 (6)	
H6A	0.2283	0.1748	1.0451	0.013*	
H6B	0.3012	0.0373	1.1161	0.013*	
C5	0.23917 (16)	−0.0678 (4)	0.97205 (19)	0.0124 (6)	
H5A	0.2842	−0.1156	0.9508	0.015*	
H5B	0.2219	−0.1721	1.0055	0.015*	
C7	0.42237 (16)	0.1000 (4)	1.04603 (19)	0.0132 (6)	
H7A	0.4613	0.1809	1.0273	0.016*	
H7B	0.4074	−0.0098	1.0003	0.016*	
C10	0.42071 (19)	0.5163 (4)	1.0745 (2)	0.0193 (7)	
H10A	0.4779	0.4604	1.1017	0.029*	
H10B	0.4218	0.6369	1.1079	0.029*	
H10C	0.4028	0.5367	1.0019	0.029*	
C1	0.05856 (19)	0.1108 (4)	0.62341 (19)	0.0215 (7)	
H1A	0.0668	0.2451	0.6151	0.032*	
H1B	−0.002	0.0793	0.5887	0.032*	
H1C	0.0922	0.0373	0.5946	0.032*	
C3	0.03637 (17)	0.0745 (4)	0.78593 (19)	0.0138 (6)	
H3	−0.0224	0.1072	0.7612	0.017*	
C4	0.08539 (16)	0.0273 (4)	0.8808 (2)	0.0134 (6)	
H4	0.0682	0.0206	0.9359	0.016*	

### Atomic displacement parameters (Å^2^)

**Table:**

	*U*^11^	*U*^22^	*U*^33^	*U*^12^	*U*^13^	*U*^23^
Cu1	0.01055 (17)	0.00944 (18)	0.01216 (18)	−0.00016 (15)	0.00545 (14)	0.00046 (14)
Cl1	0.0154 (4)	0.0136 (4)	0.0110 (3)	0.0043 (3)	0.0068 (3)	0.0018 (3)
Cl2	0.0162 (4)	0.0190 (4)	0.0114 (3)	0.0037 (3)	0.0048 (3)	0.0018 (3)
Cl3	0.0113 (3)	0.0140 (4)	0.0119 (3)	0.0003 (3)	0.0051 (3)	0.0012 (3)
Cl4	0.0185 (4)	0.0115 (4)	0.0420 (5)	−0.0021 (3)	0.0196 (4)	−0.0007 (3)
N3	0.0081 (12)	0.0116 (12)	0.0062 (11)	0.0000 (10)	0.0020 (10)	0.0009 (9)
N2	0.0094 (12)	0.0063 (13)	0.0093 (11)	−0.0013 (9)	0.0017 (10)	−0.0026 (9)
N1	0.0130 (12)	0.0092 (13)	0.0098 (12)	−0.0028 (10)	0.0007 (10)	−0.0026 (9)
C9	0.0158 (15)	0.0119 (15)	0.0110 (14)	−0.0007 (12)	0.0049 (12)	−0.0017 (11)
C2	0.0153 (15)	0.0090 (15)	0.0138 (15)	−0.0033 (12)	0.0063 (13)	−0.0046 (11)
C8	0.0107 (14)	0.0169 (16)	0.0182 (15)	0.0005 (13)	0.0020 (12)	0.0005 (13)
C6	0.0070 (14)	0.0142 (16)	0.0101 (14)	−0.0004 (11)	0.0029 (12)	0.0007 (11)
C5	0.0095 (14)	0.0109 (16)	0.0163 (15)	0.0002 (12)	0.0045 (12)	0.0023 (12)
C7	0.0092 (14)	0.0142 (16)	0.0184 (15)	0.0006 (12)	0.0079 (12)	0.0011 (12)
C10	0.0273 (17)	0.0147 (17)	0.0151 (15)	−0.0083 (13)	0.0075 (13)	−0.0039 (12)
C1	0.0277 (18)	0.0217 (18)	0.0119 (15)	0.0021 (14)	0.0044 (14)	0.0009 (13)
C3	0.0098 (14)	0.0136 (17)	0.0179 (15)	−0.0019 (12)	0.0055 (13)	−0.0039 (12)
C4	0.0106 (14)	0.0146 (15)	0.0165 (15)	−0.0034 (12)	0.0070 (13)	−0.0037 (12)

### Geometric parameters (Å, º)

**Table:**

Cu1—Cl4	2.2267 (7)	C8—H8A	0.98
Cu1—Cl3	2.2447 (6)	C8—H8B	0.98
Cu1—Cl2	2.2456 (8)	C8—H8C	0.98
Cu1—Cl1	2.2644 (7)	C6—C5	1.527 (4)
N3—C6	1.499 (3)	C6—H6A	0.99
N3—C7	1.507 (3)	C6—H6B	0.99
N3—C9	1.509 (3)	C5—H5A	0.99
N3—H3N	0.93	C5—H5B	0.99
N2—C2	1.324 (3)	C7—H7A	0.99
N2—C4	1.379 (3)	C7—H7B	0.99
N2—C5	1.458 (3)	C10—H10A	0.98
N1—C2	1.329 (3)	C10—H10B	0.98
N1—C3	1.374 (3)	C10—H10C	0.98
N1—C1	1.463 (3)	C1—H1A	0.98
C9—C10	1.513 (4)	C1—H1B	0.98
C9—H9A	0.99	C1—H1C	0.98
C9—H9B	0.99	C3—C4	1.337 (4)
C2—H2	0.95	C3—H3	0.95
C8—C7	1.508 (4)	C4—H4	0.95
			
Cl4—Cu1—Cl3	134.47 (3)	C5—C6—H6A	108.6
Cl4—Cu1—Cl2	99.16 (3)	N3—C6—H6B	108.6
Cl3—Cu1—Cl2	100.69 (3)	C5—C6—H6B	108.6
Cl4—Cu1—Cl1	97.04 (3)	H6A—C6—H6B	107.5
Cl3—Cu1—Cl1	98.33 (3)	N2—C5—C6	112.7 (2)
Cl2—Cu1—Cl1	133.31 (3)	N2—C5—H5A	109
C6—N3—C7	112.7 (2)	C6—C5—H5A	109
C6—N3—C9	109.68 (18)	N2—C5—H5B	109
C7—N3—C9	113.2 (2)	C6—C5—H5B	109
C6—N3—H3N	107	H5A—C5—H5B	107.8
C7—N3—H3N	107	N3—C7—C8	113.7 (2)
C9—N3—H3N	107	N3—C7—H7A	108.8
C2—N2—C4	108.8 (2)	C8—C7—H7A	108.8
C2—N2—C5	126.0 (2)	N3—C7—H7B	108.8
C4—N2—C5	125.19 (19)	C8—C7—H7B	108.8
C2—N1—C3	108.4 (2)	H7A—C7—H7B	107.7
C2—N1—C1	125.8 (2)	C9—C10—H10A	109.5
C3—N1—C1	125.9 (2)	C9—C10—H10B	109.5
N3—C9—C10	112.5 (2)	H10A—C10—H10B	109.5
N3—C9—H9A	109.1	C9—C10—H10C	109.5
C10—C9—H9A	109.1	H10A—C10—H10C	109.5
N3—C9—H9B	109.1	H10B—C10—H10C	109.5
C10—C9—H9B	109.1	N1—C1—H1A	109.5
H9A—C9—H9B	107.8	N1—C1—H1B	109.5
N2—C2—N1	108.4 (2)	H1A—C1—H1B	109.5
N2—C2—H2	125.8	N1—C1—H1C	109.5
N1—C2—H2	125.8	H1A—C1—H1C	109.5
C7—C8—H8A	109.5	H1B—C1—H1C	109.5
C7—C8—H8B	109.5	C4—C3—N1	107.6 (2)
H8A—C8—H8B	109.5	C4—C3—H3	126.2
C7—C8—H8C	109.5	N1—C3—H3	126.2
H8A—C8—H8C	109.5	C3—C4—N2	106.8 (2)
H8B—C8—H8C	109.5	C3—C4—H4	126.6
N3—C6—C5	114.8 (2)	N2—C4—H4	126.6
N3—C6—H6A	108.6		
			
C6—N3—C9—C10	−176.3 (2)	C4—N2—C5—C6	−72.8 (3)
C7—N3—C9—C10	56.9 (3)	N3—C6—C5—N2	−88.4 (3)
C4—N2—C2—N1	−0.5 (3)	C6—N3—C7—C8	−63.9 (3)
C5—N2—C2—N1	179.5 (2)	C9—N3—C7—C8	61.3 (3)
C3—N1—C2—N2	0.4 (3)	C2—N1—C3—C4	−0.1 (3)
C1—N1—C2—N2	179.5 (2)	C1—N1—C3—C4	−179.2 (2)
C7—N3—C6—C5	−65.8 (3)	N1—C3—C4—N2	−0.2 (3)
C9—N3—C6—C5	167.1 (2)	C2—N2—C4—C3	0.4 (3)
C2—N2—C5—C6	107.2 (3)	C5—N2—C4—C3	−179.5 (2)

### Hydrogen-bond geometry (Å, º)

**Table:**

*D*—H···*A*	*D*—H	H···*A*	*D*···*A*	*D*—H···*A*
N3—H3*N*···Cl1	0.93	2.29	3.134 (2)	150
N3—H3*N*···Cl3	0.93	2.79	3.399 (2)	124
C2—H2···Cl4^i^	0.95	2.66	3.480 (3)	145
C3—H3···Cl2^ii^	0.95	2.75	3.423 (3)	128
C3—H3···Cl3^ii^	0.95	2.77	3.537 (3)	138
C4—H4···Cl2^iii^	0.95	2.84	3.608 (3)	139
C9—H9*A*···Cl1^iii^	0.99	2.78	3.617 (3)	143

Symmetry codes: (i) *x*, *y*−1, *z*; (ii) −*x*, *y*−1/2, −*z*+3/2; (iii) *x*, −*y*+1/2, *z*+1/2.
