# Supplementary figures and images for: Crystal structure of 1-[2-(di­ethyl­aza­n­ium­yl)eth­yl]-3-methyl­imidazolium tetra­chlorido­cuprate(II)
Source: Acta Crystallogr E Crystallogr Commun. 2015 Apr 18;71(Pt 5):m110–1. doi: 10.1107/S2056989015006799 (PMC4420035; doi:10.1107/S2056989015006799)

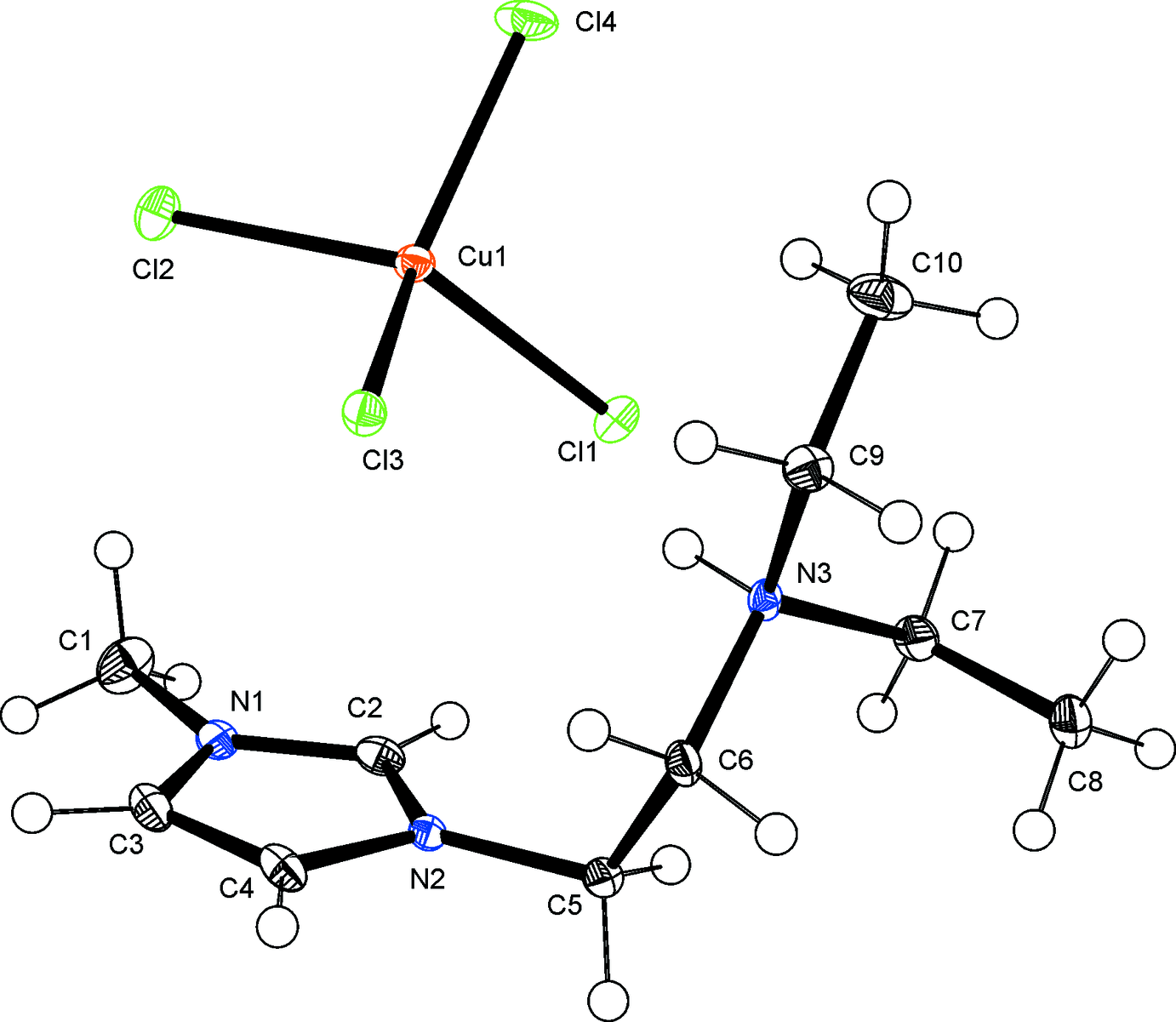

Supplement: Supplementary file 5 [file e-71-0m110-fig1.tif]

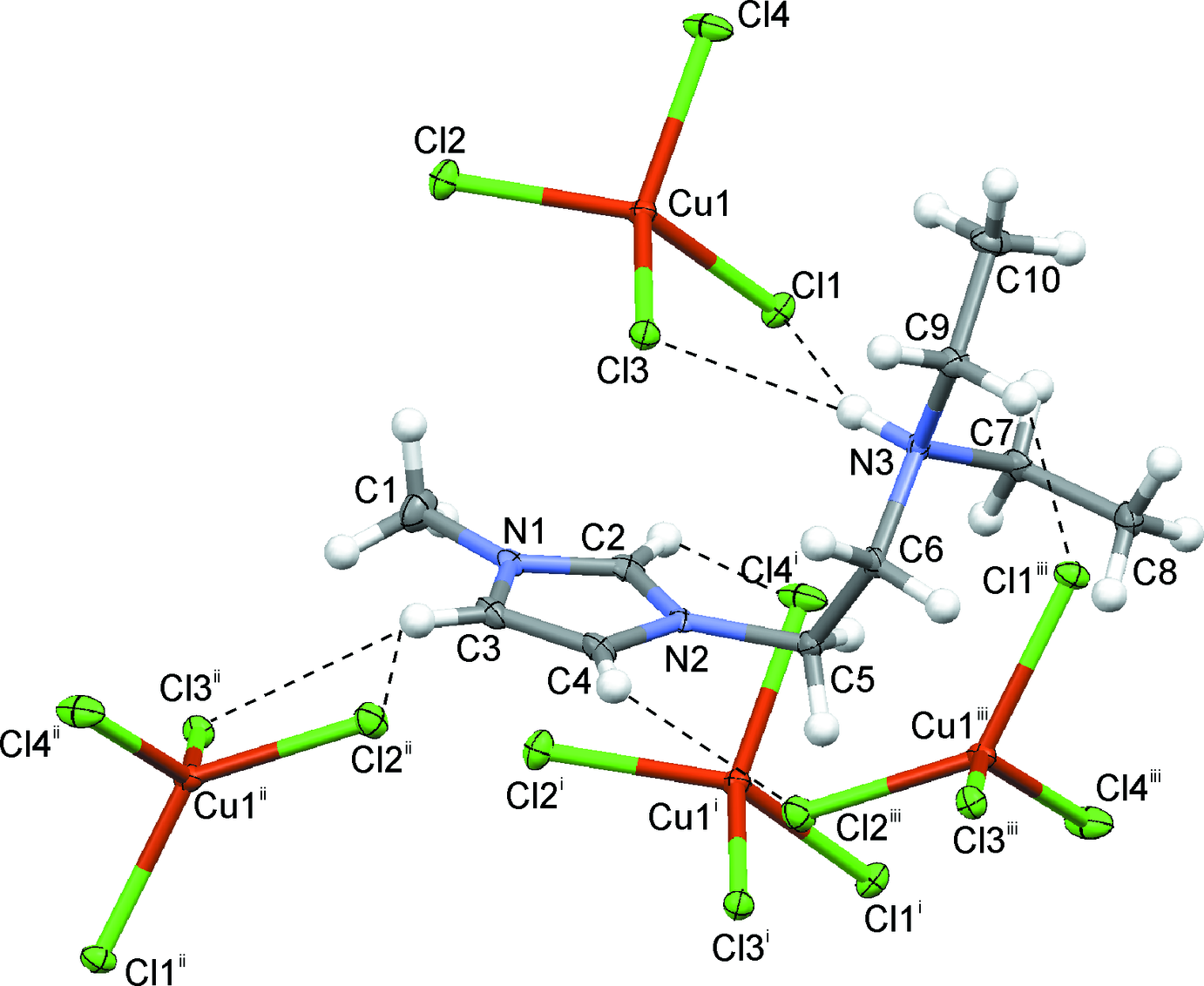

Supplement: Supplementary file 6 [file e-71-0m110-fig2.tif]
